# Sleep Promotion among Critically Ill Patients: Earplugs/Eye Mask versus Ocean Sound—A Randomized Controlled Trial Study

**DOI:** 10.1155/2020/8898172

**Published:** 2020-12-23

**Authors:** Abhilasha Chaudhary, Vinay Kumari, Neetu Neetu

**Affiliations:** ^1^Department of Nursing, Nepal Medical College and Teaching Hospital, Kathmandu, Nepal; ^2^Chitkara School of Health Sciences, Chitkara University, Punjab, India; ^3^Extendicare Maple View, Sault Ste Marie, Ontario, Canada

## Abstract

**Background:**

Poor sleep quality is common in the intensive care unit (ICU), where several factors including environmental factors contribute to sleep deprivation.

**Objective:**

This study aims to assess and compare the effectiveness of earplugs and eye mask versus ocean sound on sleep quality among ICU patients.

**Design:**

A true experimental crossover design was used. *Setting*. Medical ICU of the Maharishi Markandeshwar Institute of Medical Sciences and Research Hospital, Mullana, India. *Participants.* Sixty-eight patients admitted in the medical ICU were randomly allocated by lottery methods into group 1 and group 2.

**Methods:**

Nocturnal nine-hour (10 : 00 pm to 7 : 00 am) for a four-night period were measured. Earplugs and eye mask versus ocean sound were crossed over between two groups. Subjective sleep quality of four nights was assessed using a structured sleep quality scale. Scores for each question range from 0 to 3, with a higher score indicating poor sleep quality.

**Results:**

Repeated measures ANOVA showed that there was a significant change in the sleep quality score (*p*=0.001), which showed that sleep quality score was improved after the administration of earplugs and eye mask and ocean sound. Fisher's LSD post hoc comparison showed a significant difference (*p*=0.001).

**Conclusion:**

Earplugs and eye mask were better than ocean sound in improving sleep quality. Earplugs, eye mask, and ocean sound are safe and cost effective, which could be used as an adjuvant to pharmacological interventions to improve sleep quality among ICU patients. However, further research in this area needs to be conducted. This trial is registered with NCT03215212.

## 1. Introduction

Sleep is an essential human need necessary for the maintenance of health, energy preservation, appearance, and physical wellbeing [1]. Enough sleep is needed for all the body systems for proper functioning, and sleep cycle disturbance can significantly impair body systems [2]. Poor sleep quality is common among patients in the intensive care unit (ICU) [2, 3]. ICU patients show reduced sleep efficiency, slow-wave, sleep fragmentation, and increased daytime sleep [4, 5]. More than 50% of critically ill patients have shown sleep disturbances [6]. Various environmental [7, 8] and nonenvironmental factors [4, 7] effect sleep in the ICU. Major environmental factors reported by Lewandowska et al. were the measurement of vital signs, light, blood collection, diagnostic tests, and noise, respectively [8]. Likewise, Lori J Delaney et al. reported noise, light, and clinical care interactions [9], whereas Ding et al. reported that psychological factors outweigh the environmental factors in contributing to poor sleep [10].

Most of the studies reported noise and light as major sleep-disrupting factors in the ICU [2, 6, 7, 11]. The 1999 World Health Organization guidelines for community noise recommend a maximum of 35 decibels (dB), adjusted for the range of normal hearing overnight and 40 dB during the day for hospital environments [12], but the noise level in the ICU ranges from 50 to 75 decibels, peaks of 85 dB. The main sources of noise are talking by staff, infusion pump alarms, monitor alarms, telephone, and television [3]. The exact mechanism of ICU noise is still debated. Noise affects sleep by causing sleep disruption [11, 13] and impairing the restorative functions of sleep [14].

Another important environmental factor causing sleep disturbance in the ICU is exposure to light [3]. Exposure to light affects the circadian rhythm. The circadian mechanism regulates the sleep by governing the variations in sleep over 24 hours, whereas the circadian rhythm is regulated by the hormone melatonin [15]. The melatonin hormone is secreted by the pineal gland. Its secretion is increased in darkness and decreased in light [16]. The melatonin secretion pattern has been related to the sleep disturbances observed in the ICU [13]. Sleep deprivation has a physiological effect on the body that may contribute to prolonged ICU stay and decreased recovery and may lead to complications such as delirium and neuropsychological problems [9]. Therefore, there is an urgent need for effective measures to promote sleep in ICU patients.

In the ICU, various pharmacological and nonpharmacological methods are used to manage the sleep disturbances. Several studies have been conducted to assess the effectiveness of nonpharmacological intervention for sleep enhancement in ICU patients [17–19]. Earplugs, eye mask, and white noise are commonly used nonpharmacological approaches for sleep promotion in the ICU. A study conducted by Fateme Mashayekhi et al. [20] showed that earplugs improved the perception and quality of sleep [20]. Similarly, a study conducted by Daneshmandi et al. [1] and Babaii et al. [21] found that eye mask enhanced sleep quality in patients of coronary care units. Additionally, many studies have been conducted administering earplugs and eye mask as a combination, and the result of the study showed positive enhancement in sleep quality among ICU patients [22–25]. Bani Younis et al. [26] reported earplugs and eye mask prolong the sleep of ICU patients, and Demoule et al. [23] reported that it reduces long awakening and increases N3 duration. White noise, which includes sounds of rain and ocean sound waves, acts as auditory masking [27]. Afsar et al. [27] reported that white noise masks environmental noises and enhances and maintains sleep. Stanchina et al. [28] reported that it increases arousal threshold, and Williamson et al. [29] reported that white noise improves depth, quality, and number of awakening.

In the past two decades, several strategies have been proposed to improve sleep in the ICU. In this study, we have administered nonpharmacological interventions, earplugs and eye mask and ocean sound. Many studies have been conducted to assess the earplugs [30] and eye mask [1, 21] effectiveness separately and white noise [27, 28] separately. However, no studies have been published yet to compare the effectiveness of earplugs and eye mask with ocean sound on sleep quality. The use of these interventions is safer and cost effective than the pharmacological method, as well as it could be used as an adjuvant to pharmacological interventions to enhance sleep quality. Using these strategies may help in further research, nursing practices, and ultimately to ICU patients. Therefore, the study aims to evaluate and compare the effectiveness of “earplugs and eye mask versus ocean sound” on sleep quality among ICU patients. The rest of the paper is organized as follows. In Section 2, we, describe the materials and methods. In Section 3, we present the results of the study. In Section 4, we describe the work. In Section 5, we conclude our work and give the recommendations.

## 2. Materials and Methods

### 2.1. Study Design and Setting

A true experimental crossover design was used in which the participants acted as their own control. The study was conducted in a 23-bedded medical ICU within the 1100-bedded Maharishi Markandeshwar Institute of Medical Science and Research Hospital, Mullana, India. The ICU was organized as two parallel rows with curtains around each bed with enough space maintained between each bed. The ICU had well-ventilated and lightening windows with curtains. Alarms, good lighting, and a continuous monitoring system were established at each patient's bedside. Patient care activities were mostly performed early morning and as per the set schedule. Sleep maintenance is one of the important aspects of care. Hence, the light was dimmed after 10.00 pm, and alarms were minimized. Television and radio were not available in the room. The Institutional Ethical Committee of Maharishi Markandeshwar University, Mullana, India, approved the study.

### 2.2. Study Participants

Study participants were recruited from November to December 2016. Participant's eligibility criteria included (a) age ≥ 18 years; (b) Glasgow coma score (GCS) > 12; (c) ability to communicate and understand the sleep questionnaires administered; (d) stable hemodynamic; and (e) length of ICU stay at the time of enrollment for, at least, 24 hours. Exclusion criteria were (a) any trauma in the head, ears, and eyes; (b) a known psychiatric illness; (c) taking sleep-inducing drugs such as narcotics and sedatives; (d) patients with sleep disorders; (e) patients who were mechanically ventilated; (e) presence of hearing impairment; (f) patients who were blind; (g) discharged during the study period; (h) patients who were unwilling to receive interventions and who discontinued intervention during the study period; and (i) patients transferred to other units from the ICU. Informed consent was taken before being enrolled in the study.

### 2.3. Randomization and Enrollment

**Figure 1:**
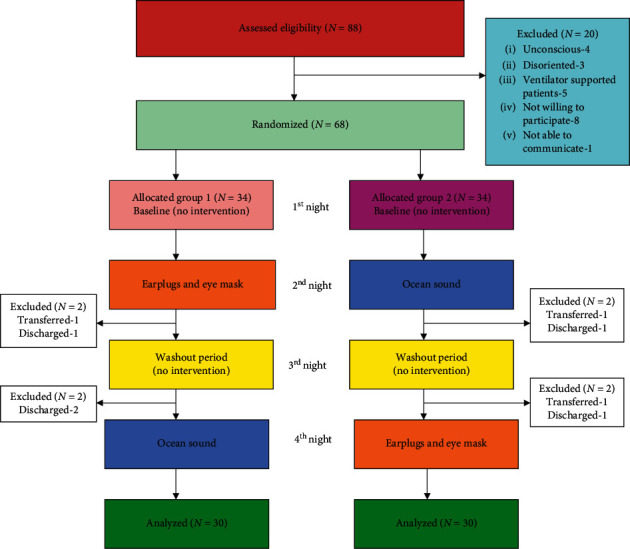
Flowchart of the study.

Participants were randomly assigned by the lottery method to two different groups, i.e., group 1 and group 2 (Figure 1). The participants with, at least, 24-hour ICU stay were enrolled in the study. The study was restricted to a four night's interval.

### 2.4. Interventions

#### 2.4.1. Earplugs and Eye Mask

Participants were instructed by the researcher to wear earplugs and eye mask. Foam earplug a made from polyurethane with noise reduction rating (NRR) = 29 dB, single number rating (SNR) = 37 dB (3M), Bengalaru- 560, India, were used. The eye mask was a delicate dark-colored cloth (black and blue) 18.5 × 8.5 cm filled with fabric and covering both eyes. Elasticized straps held the mask in place and prevented incoming light and allowed a state of pure darkness. The researcher offered earplugs and eye mask to the participants from 10 : 00 pm to 7 : 00 am.

#### 2.4.2. Ocean Sound

Ocean sound is a type of white noise, which is a soothing sound of ocean waves crashing on the beach, which was provided by the researcher via earphones for 30 minutes during the onset of sleep from 9 : 15 pm to 9 : 45 pm. It was provided via the android mobile (HTC One 4.4.2) application, namely, relax melodies meditation of Ipnos software.

### 2.5. Assessment

#### 2.5.1. Baseline Data

Demographic data and factors affecting sleep before hospitalization and in the ICU were collected by a face to face interview method following written informed consent after the first night. Demographic data included age, gender, marital status, religion, the main reason for ICU admission, duration of hospitalization, and previous history of hospitalization.

#### 2.5.2. Outcome

Subjective sleep quality was the primary outcome. Participants had difficulty in understanding and comprehending the Pittsburgh Sleep Quality Index (PSQI) [31] scale during the pretest. Hence, a structured sleep quality scale was developed by the researcher modifying the PSQI and based on reviewing the Richards Campbell Sleep Questionnaire (RCSQ), Verran Synder Halpern sleep scale (VSH) [32]. Content validation of the questionnaire was performed by seven experts from the field of nursing, medicine, and psychiatry. Content validity was calculated as the scale content validity index (S-CVI) = 0.87, and the reliability was 0.85. It consisted of 11 self-reported items where one of the items had 1 subitem. The items of the scale were sleep quality, sleep depth, sleep latency, awakening, returning to sleep, noise, light, sleep duration, status of freshness, disturbances in daily activities, and sleep efficiency. Scores for all 11 questions range from 0 to 3 (0 = very good, 1 = fairly good, 2 = fairly bad, and 3 = very bad), whereas, those of 1 subitem question range from 0 to 1 (0 = very good and 1 = fairly good). The total score was calculated by finding the sum of the 11 items, and the total score ranges from 0 to 34. A higher score indicated poor sleep quality. The researcher evaluated the subjective sleep quality for four nights, every morning at 7 : 30 a.m.

#### 2.5.3. Participants Acceptability

The acceptability level of earplugs, eye mask, and ocean sound was assessed by a semantic differential scale every morning after administering the intervention. Respondents were asked to rate the acceptability on a series of bipolar adjectives. Scores 1 to 5 were assigned to each bipolar scale responses, where 1 = lowest acceptability and 5 = highest acceptability.

### 2.6. Procedure of Data Collection

During the first night following randomization, no intervention was given to both groups and acted as a baseline. On the second night, group 1 participants received earplugs and eye mask and group 2 participants received ocean sound. A one-day gap was kept to reduce the carryover effect; hence, no intervention was provided to both the groups on the third night. On the fourth night, interventions were crossed over, group 1 participants received ocean sound and group 2 participants received earplugs and eye mask. Sleep quality was assessed every morning at 7 : 30 a.m. for four nights using a structured sleep quality scale by the researcher.

### 2.7. Statistical Analysis and Sample Size Calculation

All data analyses were performed using SPSS version 16.00. Baseline characteristics and factors affecting sleep among both the groups were assessed using the chi-square test (for categorical variables) and independent *“t'*-”test (for a continuous variable with a normal distribution). Sleep quality before the intervention was assessed using an independent *“t*-*”*test. To rule out the carryover effect, the paired *‘t'*-test was used. The difference in the mean sleep quality score at four time points was compared using Repeated Measures Analysis of Variance (ANOVA). Fisher's Least Significant Difference (LSD) post hoc test was used to determine which specified means differ during the four nights of interventions. *p* < 0.05 was considered significant.

The sample size was calculated based on the findings of an earlier study [33] which found that the mean sleep quality score was 23.7 ± 20.6 and 54.0 ± 25.5 in the intervention (*n* = 20) and control group (*n* = 25) among ICU patients. Using the following data, an effect size of 1.307 was calculated, and the sample size required for the study was determined.

(1) $$\begin{array}{c} \text{Cohen's d }\left(\text{effect size}\right)=\frac{{\text{Mean}}_{\text{control}}{-\text{Mean}}_{\text{Experimental}}}{\text{Pooled S.D}},\\ =\frac{54-23.7}{23.17},\\ =1.30. \end{array}$$

Considering 1.30 as the effect size, alpha as 0.05, and beta as 0.2, the sample size was calculated to be 10 for each group. Considering dropouts, a sample size of 34 in each group was recruited for the study.

## 3. Results

Participants were screened between November and December 2016. In total, 68 patients who met the inclusion criteria were enrolled and randomly divided into two groups, with 34 in each group. Eight participants were excluded after randomization, four in each group due to discontinuation of the intervention during the study, i.e., in group 1, discharged (*N* = 3) and transferred to another unit (*N* = 1) and in group 2, discharged (*N* = 2) and transferred to another unit (*N* = 2). Thus, data analyses were carried out for 30 in each group (Figure 1). As illustrated in Table 1, study participants were predominately male (51.7%) and female (48.3%) with an average age of (51.2 ± 14.8) years. The majority of the participants was married (71.6%) and followed the Hindu (66.6%) religion. The main reason for ICU admission was due to cardiovascular (46.6%) and respiratory (28.3%) problems. More than half of the participants (55%) had a previous history of hospitalization. The mean length of ICU stay at the time of enrollment was 2.23 ± 0.56 days. The two study groups were homogenous and comparable in terms of age, gender, marital status, religion, main reason for ICU admission, previous history of hospitalization, and length of ICU stay.

**Figure 2:**
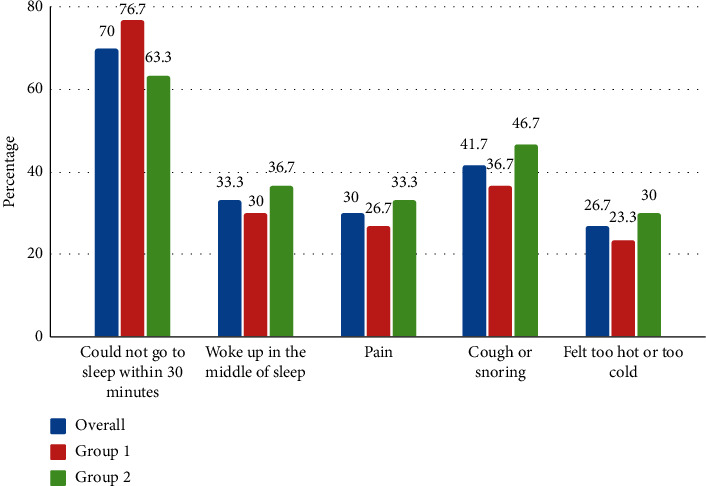
Factors affecting sleep at home before hospitalization.

**Figure 3:**
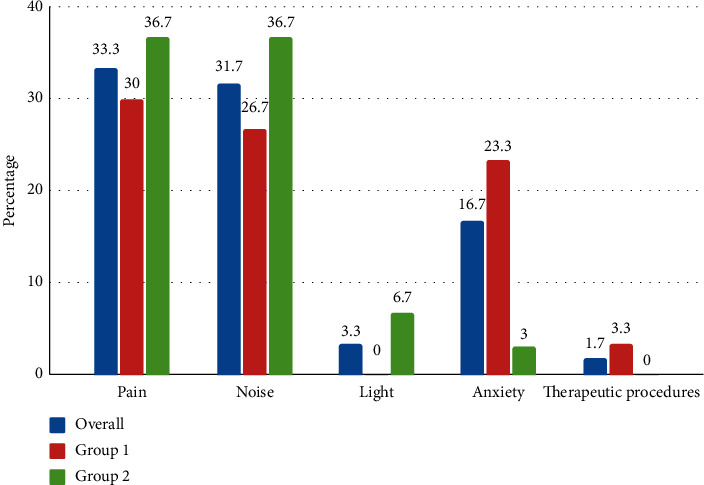
Factors affecting sleep in the ICU.

Table 2 shows the factors affecting sleep at home (Figure 2) before hospitalization and in the ICU (Figure 3). Most of the participants (76.7%) reported the presence of sleep disturbances in the ICU. Sleep-disturbing factors reported in the ICU were pain (33.3%), noise (31.7%), anxiety (16.7%), and light (3.3%). Sleep-disturbing factors reported at home were could not go to bed within 30 minutes (70%) and pain (18%).

As shown in Table 3, the difference in sleep quality score was found to be statistically nonsignificant which showed that both the groups were similar and comparable in terms of sleep quality score before administration of intervention.

### 3.1. Carryover Effect

Third night was kept as a washout period where no intervention was provided in both the groups. There was no significant difference (*p*=0.08) in the mean sleep quality score after the 1^st^ and 3^rd^ night which showed that there was no carryover effect of one intervention on the other (Table 4).

### 3.2. Primary Outcome (Sleep Quality)

Results of the sleep quality score among group 1 and group 2 at four time points, i.e., after the 1^st^ night, 2^nd^ night, 3^rd^ night, and 4^th^ night, respectively, are shown in Table 5 and Table 6. Repeated measures ANOVA showed that there was a significant change in the sleep quality score (*p*=0.001), which showed that the sleep quality score was improved after the administration of earplugs and eye mask and ocean sound among both the groups. A further post hoc test was applied to see at what time point the difference in the sleep quality score was significant. The result of post hoc comparison is shown in Table 7 and Table 8 which depicted that a significant difference was observed (*p*=0.001) and showed that earplugs and eye mask were found to be more effective than ocean sound among both the groups.

### 3.3. Acceptability Level of Earplugs, Eye Mask, and Ocean Sound

Participants' acceptability level of earplugs, eye mask, and ocean sound are listed in Table 9. Overall, most of the participants had high acceptability, i.e., earplugs (95%), eye mask (98.3%), and ocean sound (93.3%).

## 4. Discussion

Consistent with the previous studies [23, 34, 35], we found that sleep quality was disturbed among ICU patients. The mean age of the participants in this study was 51.2 ± 14 years which is consistent with the findings of Simon et al. (63.9 years) [14] and Mashayekh et al. ((51 ± 18)) years [20], which shows that majority of the participants admitted in the ICU are of middle adulthood. In this study, eight participants were excluded during the study period because of being discharged and transferred to other units. We have excluded those participants as we have kept it as exclusion criteria because we have limited the sleep assessment in the ICU setting only. The majority of the participants in this study were married and followed the Hindu religion. Though we have not found the relationship of sleep with marital status and religion, few studies reported these variables are related to sleep. The study conducted by Lauren Hale showed that unmarried individuals were more likely to sleep for a short duration compared to married people [36]. A study conducted by Michael A. Grandner et al. reported that divorced people faced more sleep problems than married and single individuals [37]. Krausel et al. reported that an individual following a religion tends to create hope which creates better sleep quality [38].

In this study, sleep-disturbing factors reported at home were could not go to bed within 30 minutes (70%), waking up in the middle of sleep (33.3%), and pain (18%) [39]. Several factors cause sleep deprivation among critically ill patients such as noise, light, care activities, staff talking, monitor alarms, infusion pump alarms, telephone and television, acute illness, and therapeutic procedure [3, 9, 11, 35]. In the present study, the majority (76.6%) of the participants reported the presence of sleep disturbance in the ICU. The main sleep-disrupting factors reported in the ICU were pain (33.3%), noise (31.7%), and light (3.3%). Stewart et al. found noise (53.6%), light (23.2%), and pain (32%) [34]. Likewise, Koushal et al. found noise (94%), light (42%), and pain (8%) [22]. The light had been reported as one of the main sleep-disturbing factors in the previous study, but in this study, only the least number of participants reported light as a sleep-disturbing factor. The reason might be because the light was dimmed during the night in ICU.

We found that the mean sleep quality score of the participants before the intervention in the ICU environment was higher, indicating poor sleep quality of the participants in both the groups. The result is similar to those reported by Hu et al. [33] and Baniyounis et al. [26]. Poor sleep quality might be due to environmental and nonenvironmental factors causing sleep disturbances. The sleep quality of the critically ill patients can be enhanced by using pharmacological (use of sedation and analgesic) and nonpharmacological (reduction in noise, light, and relaxation technique) approaches [26].

In this study, we have used a nonpharmacological method such as earplugs, eye mask, and ocean sound to improve sleep quality. Our goal in this study was to assess and compare the effectiveness of earplugs and eye mask versus ocean sound on sleep quality among ICU patients. A study was conducted by Hu et al. to determine the effectiveness of earplugs and eye mask on sleep. The study period was for four-night intervals only, and the result showed ear plug and eye mask effectively promoting sleep quality [13]. Hence, we also limit our study period to 4 nights only. In this study, sleep quality was significantly improved after the use of earplugs and eye mask in both groups. This result is supported by the findings of previous studies [22, 33, 40]. The interpretation of the improvement in sleep quality after the use of eye mask arises from circadian rhythm, which is the important regulatory mechanism of sleep. Melatonin is a vital hormone, and its secretion regulates the circadian cycle. The secretion of melatonin is maximum at night in absence of light, which helps in sleep promotion [9, 41]. The use of eye mask decreases the intensity of light and creates darkness, which enhances melatonin secretion and leads to sleep arousal and maintenance. On the other hand, the use of earplugs decreases auditory perceptual processing and prevents sleep arousal.

Many studies have been conducted to assess the effectiveness of white noise on sleep quality [17, 27, 28]. The sound of rain and ocean waves was the most used white noise [27]. In this study, we have administered ocean sound to the patients. In this study, sleep quality was significantly improved after the use of ocean sound, which is in line with the study conducted by Williamson et al. [29]. A study conducted by Stanchina et al. showed that a combination of white noise and recorded ICU noise improved sleep quality [28]. Likewise, Pouya et al. showed that white noise improved sleep quality [27]. The mechanism of white noise to improve sleep quality is white noise inhibits the intense auditory stimuli stimulation to the cerebral cortex during sleep by increasing the hearing threshold level to its highest rate leading to a decrease in auditory perceptual processing [27, 28]. Besides, white noise may induce participants to habituate to the sounds in the recording, leading to sleep arousal [28].

In this study, earplugs, eye mask, and ocean sound had remarkably improved sleep quality among ICU patients. On comparing the effectiveness, earplugs and eye mask were found to be more effective than ocean sound. No studies have been conducted yet comparing these interventions. The variation in outcome might be because, firstly, eye mask and earplug are two interventions given simultaneously, whereas ocean sound was a single intervention. Secondly, ocean sound was administered for 30 minutes only, whereas the earplugs and eye mask were administered for nine hours. Ocean sound was given for less time because the patient felt discomfort for a longer duration.

In the present study, a crossover design was used which may create the possibility of carryover effect. The third night in this was study kept as a washout period to reduce the carryover effect. In the present study, there was no significant difference in the mean sleep quality score after the 1^st^ and 3^rd^ night, which showed that there was no carryover effect of one intervention on the other. These findings are in line with those of Koushal et al. which showed that there was no significant carryover effect (*p*=0.085) of the first night interventions on the second night [22].

In this study, despite the small sample size, we found the significant effect of the intervention on sleep which might be because of the strong design randomized controlled trial crossover design. A study conducted by Krogh et al. emphasized that the advantage of the crossover design is the need for fewer participants and resource consumption [42]. Similarly, previous studies with less sample size have found a significant effect on sleep quality [13, 15, 23, 29].

Acceptability of these interventions is very important. The majority of the participants in Hu et al.'s study [13] reported the earplugs and eye mask to be very comfortable, helpful, and easy to use. In contrast, ICU patients found earplugs and eye mask uncomfortable [43]. However, in this study, the majority of participants had high acceptability for earplugs, eye mask, and ocean sound. The higher acceptability might be because all the participants who did not like or not willing to use those interventions were excluded before the study as it was one of the exclusion criteria. However, few participants in this study reported earplugs and eye mask to be uncomfortable, tight, and cause difficulty while applying. Similarly, few participants had reported ocean sound as an uncomfortable and irritating sound.

### 4.1. Limitations of the Study

This study has a few limitations, which should be noticed. First, this study included a small sample size and a single setting only which limits the power of statistical analyses, and the result cannot be generalized to the population and all settings. Second, the self-report technique was used, which only evaluated subjective sleep quality and did not determine the objective sleep quality. Although polysomnography is a commonly used standard procedure for sleep measurement, it was not used because of technical difficulties, high cost, and feasibility. Third, the study only assessed a nine-hour nocturnal period rather than a 24-hour period. Therefore, further research should focus on measuring sleep patterns over 24 hours. Fourth, the study period was limited to four nights only. Fifth, ocean sound was administered for 30 minutes only as the participants reported discomfort on administering for a longer period, and this may have created variation in its outcome. Sixth, all the medications used by the patients having a probable effect on sleep were not studied and controlled in this study. Seventh, researcher bias was not controlled as the researcher had provided the intervention and assessed the sleep quality.

## 5. Conclusions

The result showed that ICU patients had poor sleep quality. Furthermore, the researcher concludes that earplugs, eye mask, and ocean sound were effective in improving sleep quality among ICU patients. Comparing the effectiveness, earplugs and eye mask were better than ocean sound in improving sleep quality. There is high acceptability for earplugs, eye mask, and ocean sound. Therefore, we recommend the use of earplugs, eye mask, and ocean sound as an adjuvant to pharmacological interventions to improve sleep quality among ICU patients. Further studies designed should consider a longer time frame for data collection, a larger sample, and observational techniques to measure sleep quality.

## Figures and Tables

**Table 1:** Baseline characteristics of participants.

Variables	Overall *N* = 60 *N* (%)	Group 1 *N* = 30 *N* (%)	Group 2 *N* = 30 *N* (%)	*p* value
*Gender*				
Male	31 (51.7)	12 (40)	19 (63.3)	0.07
Female	29 (48.3)	18 (60)	11 (36.7)	

Age (years, mean ± SD)	51.2 ± 14.8	52.7 ± 12.37	49.7 ± 16.98	0.43

*Marital status*				
Married	43 (71.6)	21 (70)	22 (73.3)	0.69
Unmarried	10 (16.6)	05 (16.7)	05 (16.7)	
Divorced	1 (1.6)	0 (0)	01 (3.3)	
Separated	1 (1.6)	01 (3.3)	0 (0)	
Widowed	5 (8.3)	03 (10)	02 (6.7)	

*Religion*				
Hindu	40 (66.6)	21 (70)	19 (63.3)	0.50
Sikh	9 (15)	04 (13.3)	05 (16.7)	
Christian	2 (3.3)	0 (0)	02 (6.7)	
Muslim	9 (15)	05 (16.7)	04 (13.3)	

*Main reason for ICU admission* ^*∗*^				
Gastrointestinal problem	14 (23.3)	10 (33.3)	04 (13.3)	0.06
Respiratory problem	17 (28.3)	9 (30)	08 (26.7)	0.71
Cardiovascular problem	28 (46.6)	09 (30)	19 (63.3)	0.10
Neurological problem	6 (10)	05 (16.7)	01 (3.3)	0.08
Urinary system problem	3 (5)	01 (3.3)	02 (6.7)	0.55
Endocrinology problem	13 (21.6)	05 (16.7)	08 (26.7)	0.34
Previous history of hospitalization	33 (55)	18 (60)	15 (50)	0.43
Length of ICU stay at the time of enrollment (days, mean ± SD)	2.23 ± 0.56	2.13 ± 0.43	2.33 ± 0.66	0.35

^*∗*^Multiple response questions.

**Table 2:** Factors affecting sleep at home before hospitalization and in ICU.

Variables	Overall *N* = 60	Group 1 *N* = 30	Group 2 *N* = 30	*p* value
	*N* (%)	*N* (%)	*N* (%)	
Presence of sleep disturbance in the ICU	46 (76.7)	22 (73.3)	24 (80)	0.43

*Factors affecting sleep in the ICU* ^*∗*^				
Pain	20 (33.3)	09 (30)	11 (36.7)	0.58
Noise	19 (31.7)	08 (26.7)	11 (36.7)	0.40
Light	2 (3.3)	0 (0)	02 (6.7)	0.15
Anxiety	10 (16.7)	07 (23.3)	03 (10)	0.16
Therapeutic procedures	1 (1.7)	01 (3.3)	0 (0)	0.31

*Factors affecting sleep at home* ^*∗*^				
Could not go to sleep within 30 min	42 (70)	23 (76.7)	19 (63.3)	0.26
Woke up in the middle of sleep	20 (33.3)	09 (30)	11 (36.7)	0.58
Pain	18 (30)	8 (26.7)	10 (33.3)	0.57
Cough or snoring	25 (41.7)	11 (36.7)	14 (46.7)	0.43
Felt too hot or too cold	16 (26.7)	07 (23.3)	09 (30)	0.68

^*∗*^Multiple response questions.

**Table 3:** Sleep quality before administration of interventions (*N* = 60).

Group	Mean ± SD	Md	SE_MD_	*t* value	^b^ *p* value
Group 1	25.23 ± 4.77	-0.74	1.29	0.56	0.57
Group 2	25.97 ± 5.26				

^b^ *p*- independent *t*-test, *‘t'*(58) = 1.67.

**Table 4:** Carryover effect in group 1 and group 2.

Group		Mean ± SD	df	*t* value	^c^ *p* value
Group 1 (*N* = 30)	1^st^ night	25.23 ± 4.7	29	1.79	0.08
	3^rd^ night	23.67 ± 2.9			

Group 2 (*N* = 30)	1^st^ night	25.97 ± 5.26	29	1.77	0.08
	3^rd^ night	24.17 ± 4.13			

^c^ *p*- paired *t*-test, ‘*t'*(29) = 2.045.

**Table 5:** Comparison of sleep quality of 4 nights among group 1 (*N* = 30).

Variable	Assessment	Mean ± SD	F value	*p* value
Sleep quality	1^st^ night	23.23 ± 4.77	73.80	0.001^*∗∗∗*^
	2^nd^ night	10.9 ± 5.06		
	3^rd^ night	23.67 ± 2.95		
	4^th^ night	16.10 ± 3.88		

^*∗∗∗*^RM ANOVA (Repeated Measures Analysis of Variance *p* < 0.001).

**Table 6:** Comparison of sleep quality of 4 nights among group 2 (*N* = 30).

Variable	Assessment	Mean ± SD	F value	*p* value
Sleep quality	1^st^ night	25.97 ± 5.26	71.59	0.001^*∗∗∗*^
	2^nd^ night	16.07 ± 4.04		
	3^rd^ night	24.17 ± 4.13		
	4^th^ night	12.43 ± 5.2		

^*∗∗∗*^RM ANOVA (Repeated Measures Analysis of Variance *p* < 0.001).

**Table 7:** Comparison of sleep quality among group 1 (*N* = 30).

Time points	Mean difference	SE_MD_	*p* value
1^st^ and 2^nd^ night 1^st^ and 3^rd^ night 1^st^ and 4^th^ night	14.33 1.5 9.1	1.46 0.87 1.14	0.001^*∗∗∗*^ 0.08 0.001^*∗∗∗*^
2^nd^ and 3^rd^ night 2^nd^ and 4^th^ night	−12.76 −5.2	1.15 0.94	0.001^*∗∗∗*^ 0.001^*∗∗∗*^
3^rd^ and 4^th^ night	7.5	5.66	0.001^*∗∗∗*^

^*∗∗∗*^Post hoc test *p* < 0.001.

**Table 8:** Comparison of sleep quality among group 2 (*N* = 30).

Time points	Mean difference	SE_MD_	*p* value
1^st^ and 2^nd^ night 1^st^ and 3^rd^ night 1^st^ and 4^th^ night	9.9 1.8 13.53	1.14 1.04 1.30	0.001^*∗∗∗*^ 0.87 0.001^*∗∗∗*^
2^nd^ and 3^rd^ night 2^nd^ and 4^th^ night	−8.1 3.6	0.89 0.98	0.001^*∗∗∗*^ 0.001^*∗∗∗*^
3^rd^ and 4^th^ night	11.73	1.09	0.001^*∗∗∗*^

^*∗∗∗*^Post hoc test *p* < 0.001.

**Table 9:** Level of acceptability of earplugs, eye mask, and ocean sound (*N* = 60).

Interventions	Low acceptability	Moderate acceptability	High acceptability
Earplugs	0 (0)	3 (5%)	57 (95%)
Eye mask	0 (0)	1 (1.6%)	59 (98.3%)
Ocean sound	1 (1.6%)	3 (5%)	56 (93.3%)

## Data Availability

The data supporting the findings of this study are available from the corresponding author upon request.
